# Exploring medical students’ perceptions and understanding of the health impacts of climate change: a qualitative content analysis

**DOI:** 10.1186/s12909-023-04769-1

**Published:** 2023-10-18

**Authors:** Ahad Heydari, Peyman Partovi, Yadolah Zarezadeh, Arezoo Yari

**Affiliations:** 1 Department of Health in Emergencies and Disasters, School of Medicine, Kurdistan University of Medical Science, Sanandaj, Iran; 2 Social Determinants of Health Research Center, Research Institute for Health Development, Kurdistan University of Medical Sciences, Sanandaj, Iran

**Keywords:** Climate change, Medical students, Healthcare system, Health impacts

## Abstract

**Background:**

Climate change has been identified as the greatest threat to global health in the twenty-first century, with its unfavorable health consequences being among its impacts on humans. Exploring the perspectives and understanding of healthcare professionals and service providers concerning climate change becomes imperative. The aim of this study is to investigate the perceptions and understanding of final-year medical students regarding the health impacts of climate change on individuals and the healthcare system using a qualitative content analysis.

**Methods:**

This study employed a qualitative content analysis approach. Face-to-face interviews were conducted with the aid of an interview guide to explore the students' awareness, understanding, and attitudes towards the impacts of climate change on public health and the healthcare system. The collected interview data were subsequently organized into codes, categories, and subcategories based on the students' perspectives and attitudes towards climate change.

**Results:**

Fifteen medical intern students were interviewed for this study, and the qualitative findings were categorized into 3 categories, 23 subcategories, and 229 codes. The study's findings revealed various health impacts of climate change, which were classified into three main categories, including environmental effects with 8 subcategories, socio-economic effects with 8 subcategories, and health effects with 7 subcategories. The study's findings revealed medical students' perceptions of various health impacts of climate change and These findings suggest that medical student understand that climate change has significant impacts on individuals' health and society, mainly through environmental degradation, increased risks, and climate-related disasters, which ultimately lead to adverse health outcomes.

**Conclusions:**

The perspectives of medical students in this study indicate that climate change may not have a direct and immediate impact on the health of individuals and communities. However, it can significantly influence their health and socio-economic well-being by exacerbating or causing environmental problems, increasing the risk of weather-related events and natural disasters, ultimately leading to adverse health outcomes. While the medical students' perspectives on the health impacts of climate change are indeed broad, incorporating scientific knowledge about this topic into the medical curriculum and educating students on how to deal with patients affected by these consequences can have a significant impact on health management. This proactive approach, despite the students' already comprehensive understanding, can enhance their preparedness to address the health effects of climate change and contribute to strengthening the healthcare system's resilience in the face of climate-related challenges.

## Background

The assessment of the negative and widespread impacts of climate change on human health and the prediction of the continuation of these consequences in the future is such that the World Health Organization has declared climate change as the "biggest threat to global health in the twenty-first century [1]. These consequences include heatwaves, cold snaps, malnutrition, and the exacerbation of cardiovascular and respiratory problems [2]. In addition, the rise of vector-borne diseases such as dengue, Zika, Chikungunya, and malaria is being increased by climate change. The direct damage caused by severe climate change events and the increased risk of unsafe water and food in areas affected by climate change are significant health impacts. Marginalized communities, children, the elderly, and those with underlying health conditions are among the vulnerable populations that are disproportionately impacted by the consequences of climate change [3].

In this regard, it is crucial to prioritize monitoring and controlling climate-related health issues like heat-related illnesses, vector-borne diseases, and cardiovascular conditions. Preparedness for unexpected events, such as floods and storms linked to climate change, is vital. Healthcare facilities should also implement preventive measures and adapt to changing climate conditions [4]. Research in the United States by Maibach EW et al. highlights that physicians are trusted sources of information regarding climate change's health impacts [5]. Another study by Sarfaty et al. indicates that most physicians recognize the connection between climate change and health and advocate for interventions to mitigate its effects [6].

Future physicians have the potential to influence patients positively in adopting healthier behaviors to reduce climate change-related health risks [7]. Educating physicians systematically has been shown to increase patient awareness of climate change and reduce its health impacts [8]. Health literacy, defined by the European Health Literacy Consortium in 2012, empowers individuals to make informed decisions related to disease prevention and health promotion, ultimately improving their quality of life [9]. To enhance society's and the healthcare system's health literacy regarding climate change's health impacts, revising medical and paramedical school curricula and fostering better attitudes among students during their education are necessary [10].

While raising awareness about climate change's global health impacts and individual responsibility is crucial, many physicians may not fully comprehend their role in addressing it [10]. Climate change significantly affects human health, posing growing dangers. Climate change experts argue that physicians, due to their trusted status, can effectively educate the public about the health impact of climate change. Consequently, it is vital to integrate climate change and its health consequences into medical education, empowering future physicians to address this issue and mitigate its effects. Such an approach enables physicians to play a pivotal role in promoting public health and safeguarding vulnerable populations from the adverse impacts of climate change [10].

Despite some studies that have evaluated the understanding of physicians and the general public regarding the health effects of climate change in relation to patient care and the role of practicing physicians [11, 12], there has been limited research [10, 11] examining the perspectives of medical students concerning the health effects of climate change and the potential contributions of medical students in mitigating these effects. Recognizing this gap, the primary objective of this study is to elucidate the attitudes and perceptions of medical students, who represent the future stewards and workforce of the healthcare system, regarding the health implications of climate change within the framework and socio-cultural context of a developing nation.

Medical students, as future healthcare professionals, must acquire the necessary knowledge and skills to address the health impacts of climate change. Through education and training programs, they can become effective advocates for climate and planetary health, and play an important role in increasing public awareness about the health impacts of climate change. By doing so, they can contribute to the development of effective public health policies and interventions to mitigate the negative impacts of climate change on human health.

## Methods

### Participants and data collection method

**Fig. 1:**
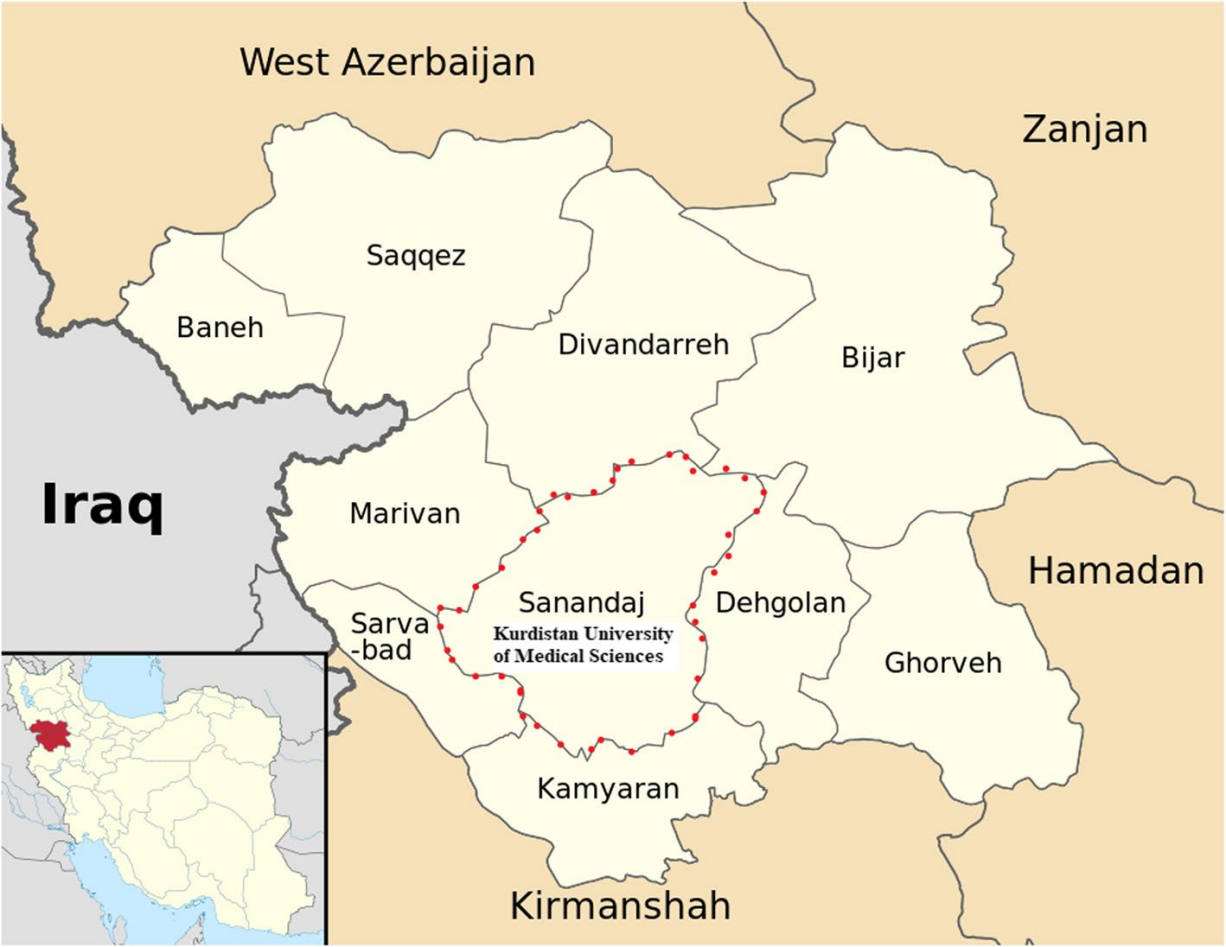
The Location of Kurdistan University of Medical Sciences in Sanandaj City, Kurdistan, Iran. Source: These maps were constructed by authors using existing data in https://commons.wikimedia.org/wiki/File:Sanandaj,_Kurdistan_Province_locator_map.svg#/media/File:Kurdistan_Province_districts_map_(with_labels).svg at 23 May 2023

For this study, face-to-face interviews were conducted with final-year medical students at Kurdistan University of Medical Sciences between May and October 2022 to collect data. Kurdistan province is located in western Iran. This province has a population of nearly 1.5 million people [13, 14]. Kurdistan University of Medical Sciences is located in the city of Sanandaj, which is in the center of this province (Fig. 1).

The study was carried out using a purposive sampling method. To this end, the researcher (PP) collected data using 15 semi-structured and non-structured face-to-face interviews. The interviews began with three non-structured interviews to determine the interview axes, followed by 12 semi-structured interviews. Before commencing the interviews, an informed consent form was presented to the students, and the interviews began after obtaining written consent. For data collection, an interview guide with open-ended questions focused on the study objectives was used. During the interviews, the researcher carefully listened to the participants' responses and asked probing questions to maintain the conversation in line with the research question. At the same time, the researcher tried to use fewer leading questions to avoid influencing the interview direction. In some cases where additional questions were needed, telephone calls were made and recorded, based on the participants' preferences. After the first interview was reviewed by one of the supervisors (AH), any issues with the interview management approach were identified and resolved in subsequent interviews. The main focus of the conversations in the interviews was the experiences and attitudes of medical students towards the health challenges of climate change. The study employed semi-open questions to investigate medical students' understanding and perspectives on climate change and its impact on health. Some of the semi-open questions used in the study were as follows:

1. What does climate change mean?
2. How can climate change affect the health of individuals and society?
3. How does climate change affect the health system?
4. How can these effects be reduced?
5. What are the solutions to increase the preparedness of the health care system for climate change?

### Data analysis

Due to the complex nature of understanding perspectives and attitudes, researchers have favored qualitative content analysis as the preferred method for extracting and categorizing students' viewpoints [15]. This method is particularly valuable when aiming to describe a phenomenon within a conceptual framework and organize the extracted data systematically. Subsequently, it allows for a conceptual examination of the phenomenon of interest [14]. In this study, the Graneheim and Lundman approach [16] was used for data analysis. The text was transcribed by PP. To make sense of the data as a whole, the text of each interview was transcribed word for word immediately after the interview, and each text was listened to and read several times by PP to immerse the researcher in the data. After identifying the meaning units and phrases in the text, condensed meaning units were extracted through note-taking by AH. Then, the participants' experiences were identified as concepts under the precise supervision of a qualitative research supervisors (AH and AY) and with the consultation of another researcher (YZ). In the next step, the initial codes were placed in subcategories based on their similarities and differences by AY and AH. Given that in the primary model, one or more subcategories were defined for each primary category, some of the initial codes were placed in the existing subcategories (AH and AY). To ensure accurate comprehension of the concepts and prevent superficial and automated coding, the coding and categorization procedures were manually conducted using paper and pencil (PP and AH). In the preliminary coding phase, the sentences of the participants were utilized, and the codes and condensed meaning units were identified. Subsequently, the codes were classified into categories and subcategories based on their similarities and differences (AY and AH).

To ensure the validity of this study and increase the trustworthiness of, four valid criteria, including credibility, transferability, dependability, and confirmability have been achieved.

In the present study, the researcher ensured credibility by being involved extensively in the study, from the beginning of the design phase to the data collection, analysis, and writing stages. In addition, the results of data analysis were shared with other qualitative researchers in this study to obtain their complementary and critical perspectives, ensuring coordination with the research team and enhancing dependability.

In this study, transferability was ensured by selecting appropriate participants who had the most interest about the research topic. Data collection and analysis were done simultaneously, and coherence between research questions and methods was ensured. Additionally, the study results were compared to those of other studies (in the discussion section) to enhance the transferability of the research. The researcher's interest in the phenomenon under study, their long-term involvement with the phenomenon, documenting interview transcripts, and efforts to obtain complementary and critical feedback from research participants, research group members, and other qualitative researchers were factors that ensured confirmability in this study.

## Results

In this study, 32 final-year medical students in the internship phase were invited to participate in the interviews, of whom 15 individuals (47%) participated in the interviews. Among the participants, 13 individuals were aged between 24 and 29 years, and 2 individuals were aged between 30 and 35 years, with a mean age of 28.8 years. The duration of the interviews ranged from 20 to 50 min, with an average of 35 min, depending on the richness of the participants' information.

Among the interviews, 229 codes were extracted and categorized into three categories (environmental, socio-economic, and health effects) and 23 subcategories. The category of environmental effects had 8 subcategories, including the effects of climate and global warming, environmental damages, effects on ecosystems, air pollution, water pollution, effects on the aquatic chain, effects on agriculture and the food chain, and weather-related hazards. The category of socio-economic effects had 8 subcategories, including population migration, education, employment, security, urban services and resources, infrastructure, vulnerable groups, and economic issues. The category of health effects had 7 subcategories, including health services, personal health, malnutrition, mental disorders, infectious diseases, vector-borne diseases, non-communicable diseases, and injuries. The extracted codes were categorized into different categories and subcategories, which are presented in Table 1. Some of the most important results related to these categories are discussed below.

**Table 1:** Categories, subcategories, and codes for the health effect of climate change

Category	Sub-Category	Code
**Environmental effects**	Global Warming Impacts	Atmospheric changes, Seasonal weather changes, Climate imbalance, Cold waves, Increase in greenhouse gases, Ozone layer depletion, Temperature inversion, Changes in air temperature, Changes in solar radiation, More absorption of sunlight, Increase in solar radiation, Increase in UV rays, Formation of heat islands, Increase the number of hot days, Heat waves
	Environmental Degradation	Submergence of islands, Dust storm, Flooding of rivers, Destruction of forests, Changes in the earth's orbit, Destruction of natural resources, Soil erosion, Increase in salt marshes
	Ecosystem	Effect on living organisms, Reduction of vegetation, Extinction of animal species, Extinction of plant species, Increase of insects, Increase of rodents, Extinction of marine creatures, Destruction of marine ecosystem, Change in native ecosystem
	Air Pollution	Increase in pollutants, Declining Air Quality, Air pollution
	Water Pollution	Groundwater pollution, Declining water quality, Reduced access to safe water, Reduction in potable water, Increase in the need for safe water, Pollution of drinking water, Decrease Environmental health of camps,
	Water Supply Chain	Reduction of underground water level, Increase in water evaporation rate, Dams reservoir reduction, annual precipitation reduction, Decrease in drinking water, Increase in non-precipitating clouds, Decrease in water absorption, Inconsistency in seasonal precipitation, Melting of glaciers, Rising sea level,Increase in stagnant water, Unusual rains, Excessive use of underground water
	Agriculture And Food Supply	Disturbance in the food chain,loss of livestock, Destruction of farming, Increase in agricultural pests, Food insecurity, Diminished agricultural products quality, Diminished livestock products quality, Decrease in agricultural products, Reduction of livestock products, Inequality in access to food sources, Loss of flowering fruit trees, Salinization of land, Food contamination
	Climatic Hazard	Drought, unusual floods, Dust storm, tsunami, Landslide
**Socio-economic**	Migration And Displacement	Urbanization, Displacement, Forced migration, Living in camps, Lack of shelter
	Education	Unmet basic needs, Educational failure, Withdrawal from education, Decreasing health literacy
	Occupation	Increase in staying at home, Increase in remote work, Destruction of businesses, Increase in unemployment, Decrease in work efficiency, Decrease in working hours, Increase in days off
	Security	Decrease in social security, Decrease in mental security, Entry of wild animals into the city,War, Increasing interpersonal tension
	Urban Services And Resource	Injustice in serving, Inequality in access to resource, Disruption of urban planning, Increased need for medical services
	Infrastructure	Destruction of houses, Fire, Destruction of health centers, Road closing, Bridges destruction, Destruction of coastal cities, Destruction of treatment plants, Destruction atomic power plants, Destruction of water treatment plants, Destruction of mines, Submergence of cities, Communication infrastructure destruction
	Vulnerable Groups	The effect on vulnerable groups, The effect on large families, The effect on the poor people, Damage on poor countries, Increase in addiction
	Economic Issue	Decrease in income, Increase in medical costs, Increase in personnel costs, Increase in reconstruction costs, Economic poverty, Decrease in purchasing power, Expensive food, Expensive livestock products, Expensive medicines, Financial losses, Lack of budget, Lack of adaptation of poor people
**Health**	Health Services	Disturbance in drug delivery, Disruption in aid delivery, Disruption in patient transfer, Diminished treatment quality, Drug shortage, Increase in workload, Decrease in access to health centers, Reducing the focus on patients, Increasing the fatigue of the treatment staff, Increasing the mental pressure of employees, Increasing the number of patients, Pandemics, Increasing diagnostic errors, Increasing the need to drugs, Lack of medical equipment, Lack of hospital beds, Lack of laboratory kits, Lack of health and medical personnel
	Personal Hygiene	Reduction of bathing, Lack of proper sanitary facilities, Reduction of oral and dental hygiene, Decreased hygiene of menstruating women, Reduction of personal hygiene
	Malnutrition	Malnutrition, Increase in global hunger, Weakening of diet, Decrease in nutrition level, Threat to food security, Reduction of mineral, Lack of vitamins, Reduced growth of children, Exophthalmia, Anemia, Scurvy, Rickets
	Mental Disorders	Anxiety disorders, Stress, Depression, Schizophrenia, PTSD, Suicide, Depression, Reduced life expectancy, Reduced interpersonal communication
	Communicable Disease	Increase in common diseases with livestock, Malt fever, Swine flu, Bird flu, Rabies, Hydatid cyst, Colds, Cholera, Gastroenteritis, Tuberculosis, Hepatitis, Tetanus, Amoebas, Increase in fungal diseases, Outbreak of new diseases, The prevalence of diseases subject to eradication
	Vector Borne Disease	Salek, Crimean Congo fever, Malaria, Lyme, Leishmania, plague, Rabbit fever
	Non- Communicable Disease	Cardiac diseases: Increased heart attack, Increased heart failure, Increased arrhythmia, Increased blood pressure Non-infectious skin diseases: Skin cancers, Sunburns, Increased lentigo, Increased skin sensitivity Lung diseases: Asthma, COPD, Lung cancer, Bronchiolitis Neonatal diseases and abnormalities: Increase in newborn malformations, Increase in congenital disorders, Increase in mental disabilities, Increase in physical disabilities, Decrease in IQ, Increase in premature births, Increase in MMR, Decrease in the body's immune system Injuries And Trauma: Frostbite, Heatstroke, Increased electrolyte disorders, Scorpion sting, Snake bite, Increased mortality, Dehydration, Increased trauma, Increase in seizures, Increase in animal bites, Increase in burns, Increase in fractures, Drowning, increase in accidents

### Environmental effects

This category included 74 codes that were classified into 8 subcategories according to Table 1. The categorized codes in this group included environmental effects and effects of climate change that have significant impacts on the Earth's climate and various regions, causing global warming, extreme weather conditions in different areas, and destruction of the environment and ecosystems. The environmental effects of climate change lead to air and water pollution, negative impacts on water and food supply chains and agriculture, and can affect the health of individuals and society. Some of the most important findings related to these subcategories are presented in the following sections.

#### Global warming impacts

One of the most significant impacts of climate change is the alteration of climate patterns, which can lead to shifts in weather patterns and atmospheric changes. Climate change also results in a rise in Earth's temperature and global warming. This is caused by changes in temperature, solar radiation, increased absorption of sunlight, heat radiation, and ultraviolet radiation due to ozone depletion. These factors contribute to the formation of heat islands, an increase in hot days, and heat waves. Interviewee 7 and 4 commented on this matter, saying, *"A rise in temperature exceeding what our bodies can tolerate can adversely affect our health. It can cause dehydration, disrupt our body's electrical system, and lead to other health issues."*

*"When I worked in the surgery department, an unusual and heavy snowfall occurred in Sanandaj city. Naturally, such events lead to an increase in fractures and traumatic injuries."*

#### Air pollution

Air pollution is a leading cause of death for millions of people worldwide each year [17, 18]. In some cases, droughts can result in the destruction of forests and wetlands, leading to desertification. As soil erosion intensifies over time, the frequency and severity of dust and sandstorms increase, causing unhealthy air for people to breathe. Interviewee 5 discussed the impact of air pollution and dust storms in Khuzestan province, Iran, saying, *"The sandstorms from Iraq cause respiratory problems such as COPD and other respiratory issues for people. These problems also contribute to an increase in cancer rates among the people of Khuzestan, making the cancer rate in this province twice as high as in other regions of the country."*

#### Water pollution

Climate change can result in increased heavy rainfall and floods, drought, and higher water temperatures, ultimately leading to changes in the quality of drinking water. These changes create new conditions for the growth of bacteria and viruses, leading to various human diseases when exposed to contaminated water. Additionally, water scarcity can also affect human health, particularly during drought conditions. Floods can also cause water pollution and limit access to drinkable water by infiltrating groundwater sources or contaminating freshwater purification systems. Interviewee 11 discussed the impact of droughts and floods on water scarcity and pollution, saying, *"Droughts result in water shortages, which have implications for public health. When there is a lack of water, people are forced to quench their thirst with unsafe water, resulting in health problems. Furthermore, floods have health effects, as they can contaminate the city's water treatment systems and lead to water pollution. Clean water supplies are affected, and the water's cleanliness is compromised."*

### Socio-economic effects

Economic and social effects were categorized into 54 codes under 8 sub-domains, as shown in Table 1. These categorized codes include the economic and social impacts of climate change on various individuals and groups.

#### Population displacement and migration

The health impact of displacement and migration due to climate change is often exacerbated when combined with other factors, such as chronic poverty and marginalization. Climate change will lead to increased urbanization as a result of increased flooding and drought, and the destruction of agricultural land. In addition, the destruction of homes and shelters due to climate change-induced disasters can lead to forced displacement and migration of individuals, which can ultimately affect their mental and physical health. Interviewee number 8 stated regarding this issue, *"Floods can cause damage and leave people under rubble, leading to casualties. The destruction of homes forces people to live in other places, such as refugee camps, where people are in closer contact with each other and have fewer sanitary facilities. They have to use public facilities which can increase the transmission of infections among people."*

#### Education

Climate change threatens children's rights to education at a global level [18]. Currently, nearly half of all children reside in countries that are highly susceptible to the effects of climate change, and the majority of these children are also exposed to vulnerable conditions. Climate events often result in damage to school infrastructure or even their destruction, which can ultimately cause children to permanently drop out of school. Also, due to forced migration, the opportunity to get an education is limited for some children. Consequently, climate change can ultimately lead to a decline in the overall literacy rate of society.

Interviewee number 3 stated regarding this issue, *"For example, in crisis-stricken areas, access to food is reduced, and people lose everything they have and are unable to meet their needs. Thus, they are forced to work to meet their own and their children's needs, and they may have to sacrifice their education. In addition, because they cannot meet their basic needs, they may not be able to meet many of their health needs. This can lead to a significant decrease in the health literacy of that region, and when the health literacy of that region decreases, diseases related to that region may increase."*

#### Infrastructure

Disasters or extreme weather events, can have destructive effects infrastructure, resulting in various consequences. The destructive effects of climate change on infrastructure can result in significant human and financial losses. Interviewee number 11 stated, *"Personal hygiene is affected even if proper health services are not available, especially in this area, where children and women are mostly affected. We know that they have physiological needs regardless of their circumstances. For example, women have their menstrual cycle and need to maintain personal hygiene. When they cannot follow these practices, they are more likely to develop certain female-related problems or diseases. Similarly, children who do not have access to proper sanitation facilities, such as bathrooms or toilets, are constantly exposed to unclean environments outside their homes, which can lead to various diseases."*

### Health effects

This category, which was extracted from the study and consisted of 101 codes, is divided into seven subcategories, including health services, personal hygiene, malnutrition, mental disorders, infectious diseases, vector-borne diseases, non-communicable diseases, and injuries. The findings related to these subcategories are presented below.

#### Health services

Climate change can lead to changes in temperature, humidity, and seasonality patterns, which can change the pattern of diseases and lead to an increase in disease burden, resulting in more visits to health centers and hospitals. Climate-related disruptions to supply chains, aid delivery, and patient transport can increase the workload and fatigue of healthcare workers, as well as an increase in casualties and patients requiring medical attention. Also, the occurrence of these disasters and climate events can lead to an increase in deaths, seizures, accidents, trauma, fractures, and drownings. Participant 3 mentions that, *“for example, an increase in diseases such as malaria, or diseases for which we have vaccines, may cause a community to lose access to vaccination, leading to an increase in disease burden and pressure on hospitals”.* Also, participant number 10 says, *"We witnessed the recent snowstorm and saw that most people who forced themselves to travel ended up in the hospital with issues such as falling, trauma, and hypothermia. In many cases, they were also faced with injuries from slipping or car accidents."*

#### Personal hygiene

Personal hygiene refers to the daily habits individuals practice for their own health, such as bathing, brushing teeth, washing hands, washing clothes, and cleaning dishes. Climate change can lead to drought and water scarcity, making it difficult to access clean and hygienic water for daily needs. On the other hand, the loss of housing and pollution of water sources due to the environmental impacts of climate change can lead to a lack of clean water for personal hygiene or even drinking purposes. Participant 10 explains, *"The loss of water resources can result in irregular bathing habits and compromised oral hygiene. Additionally, floods can disrupt water and sewage systems, leading to an increase in infectious diseases that were previously uncommon among the population."*

#### Malnutrition

In this study, malnutrition refers to individuals' inability to access sufficient water and food to meet their daily needs due to the effects of climate change. With water insecurity and the prevalence of climate change-related diseases, storms create a perfect storm for unprecedented global nutrition crises. Malnutrition leads to a decrease in vitamins and minerals in the body, which can result in stunted growth in children, kwashiorkor, anemia, scurvy, and rickets.

Participant 12 explains, *"The lack of access to food and the increase in food prices due to climate change is causing certain communities to have less access to food. This leads to a smaller dietary intake, malnutrition, and health problems that put their well-being at risk".*

#### Mental disorders

Climate change and global warming can also lead to an increase in forced migration, which can lead to stress, depression, and anxiety disorders. The rise in climate change-related disasters can result in mental health issues such as post-traumatic stress disorder, adjustment disorder, and depression. Additionally, it can contribute to an increase in physical illnesses that are often accompanied by psychological distress. Heat waves and high temperatures can also cause mood disorders, anxiety disorders, cognitive decline, and social anxiety disorders. Participant 14 states, *"The increasing heat and destruction of homes can lead to psychological damage, such as obsessive–compulsive disorders, anxiety disorders, and post-traumatic stress disorder. It can also increase the risk of suicide".*

#### Communicable disease

The increase in temperature and the decrease in access to clean water, as well as the increase in the population of insects such as mosquitoes, ticks, and cockroaches, can lead to an increase in waterborne infections such as cholera, amoebiasis, and gastroenteritis. Participant number 3 says, *"Due to the shortage of water or lack of access to purified water, some people are forced to use stagnant water. Stagnant water contains various microorganisms such as cholera and amoebiasis, which can cause severe diarrhea, vomiting, and other diseases related to those infections."*

#### Vector-borne diseases

The term "vector-borne diseases" in this study refers to the diseases that are transmitted through the increase in the population of vectors due to changes in seasonal patterns, temperature, and humidity. Increased rainfall can increase the amount of stagnant water and create more breeding grounds for many vectors. Droughts can also create suitable conditions for vector breeding by forming pools of stagnant water. Participant number 9 says, *"Water sources that were previously flowing like springs are now becoming stagnant, which increases the risk of parasitic diseases. The population of mosquitoes, ticks, and other vectors also increases, which in turn increases the risk of certain diseases."*

#### Non-communicable diseases

The term "non-communicable diseases" refers to cardiovascular diseases that are exacerbated by the effects of climate change, including severe heat waves, dust storms, increased pollution, and decreased air quality. Some of these non-communicable diseases, including cardiovascular diseases such as heart attacks, heart failure, arrhythmias, and hypertension, as well as some cancers, respiratory diseases, mental disorders, trauma, and malnutrition, are likely to increase in frequency and severity due to the effects of climate change, especially in individuals with underlying conditions. Participant number 3 states: *"Sandstorms can cause new respiratory diseases that the area has not experienced before and exacerbate asthma. Increased pollution can also jeopardize heart health by increasing the incidence of heart attacks, arrhythmias, and heart failure."*

## Discussion

**Fig. 2:**
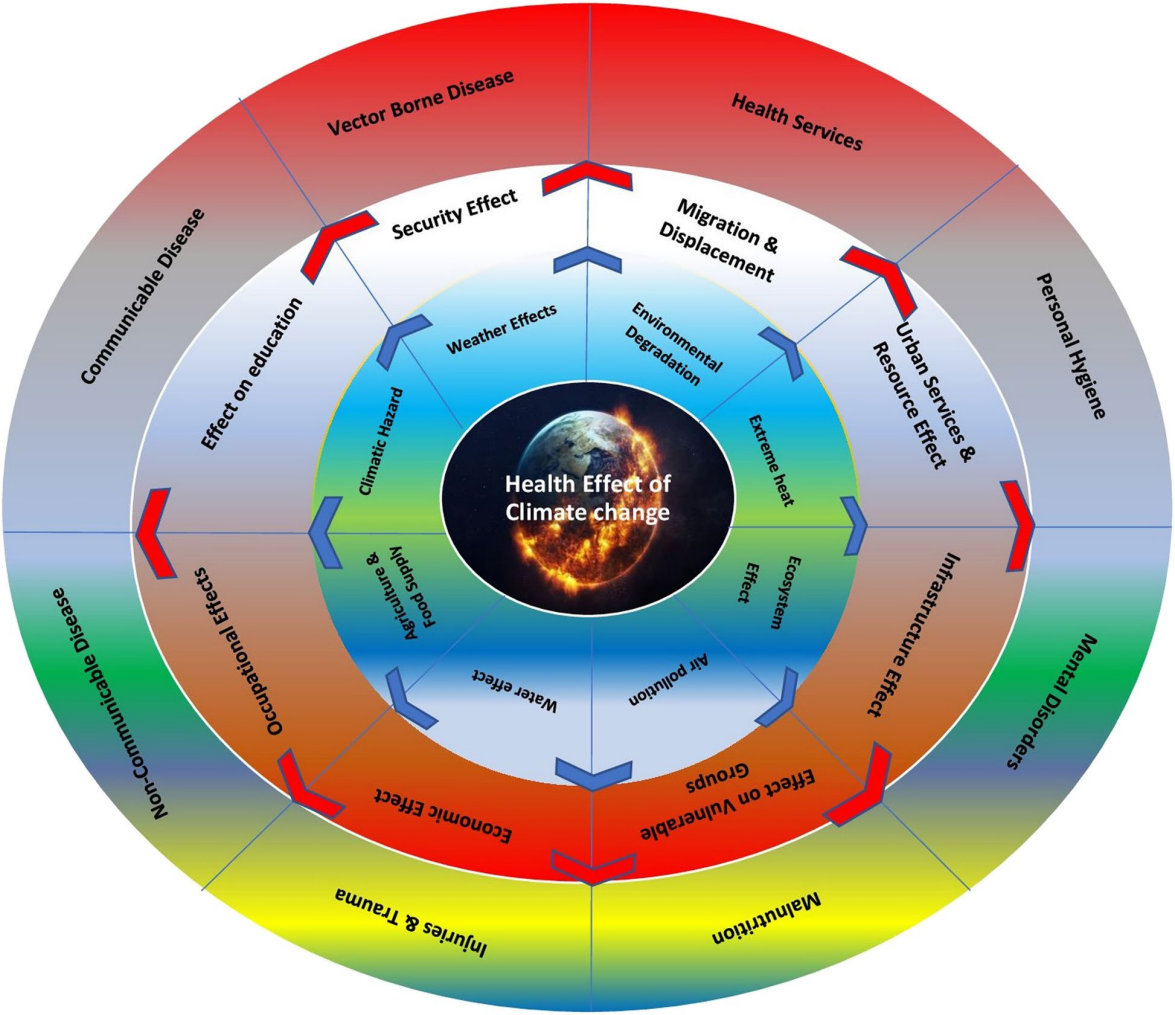
Direct and Indirect Effects of Climate Change on Health Our figure is based on the concept from the CDC source and has been customized to suit the specific content of this study Adapted from a concept inspired by a CDC figure: https://www.cdc.gov/climateandhealth/effects/default.htm

Climate change impacts health and wellbeing [19]. These impacts are extensive, with some recent emergence. Investigating these challenges is crucial for public health adaptation [4]. Assessing medical students' knowledge is valuable. A November 2021 survey found that 69% of physicians, 67% of clinical leaders, and 54% of executive managers understand climate change's health impacts [20]. This study, from the perspective of medical students, highlights their deep understanding and identifies unique health outcomes from climate change through content analysis (Fig. 2).

### Environmental effect

The comprehensive discussions held by the study participants have enriched the evidence base by providing nuanced insights into how climate change impacts health through its influence on the biophysical environment. This study offers a more comprehensive perspective compared to a similar study conducted by Sorgho and colleagues in Germany [21]. While Sorgho's study primarily focused on climate hazards and the direct and indirect health effects of climate change, our study encompasses climate hazards as one of several subcategories within environmental impacts. Similarly, Ebi and colleagues [22] concentrated on health impacts of weather-related events and climate change. However, it's important to note that climate change affects health through its influence on the biophysical environment [23]. Fundamental health necessities such as clean air, water, food, and shelter are affected [24].

Medical students dedicated have systematically identified a diverse range of health-related consequences attributed to factors, from rising temperatures and droughts to sea-level rise, flooding, wildfires, air quality degradation, and water supply challenges. These multifaceted effects significantly impact human health, leading to increased mortality rates and psychological well-being concerns [22]. Notably, extreme heat events (EHEs) are particularly lethal [25], with elevated temperatures contributing to heat-related illnesses and fatalities, accounting for over a third of such deaths in the last 30 years [26]. The IPCC predicts a concerning doubling of heat events every six years with a 1.5°C global warming scenario, a rate ten times faster than historical records [25]. Vulnerable populations are disproportionately affected [27].

Climate change affects water resources and public health through reduced access to clean water, increased infectious disease risks, and threats to coastal communities and industries due to rising sea levels. Intense precipitation raises the risk of flooding and casualties [28]. Medical students in this study have emphasized the health impacts of environmental degradation and climate-induced ecosystem changes, often overlooked. It is conceivable that the attention of medical students to this issue stems from the fact that high environmental degradation levels correlate with lower life expectancies and higher infant mortality rates [29]. Prioritizing actions to mitigate climate change's health effects by addressing environmental impacts and safeguarding ecosystems is recommended. These findings underscore the urgency of addressing climate-related health risks comprehensively and emphasize the need for immediate action to protect the well-being of all communities.

### Socio-economic effect

While medical students are well-informed about the impact of economic and social factors on health, our study extensively explores the effects of climate change on health, especially in these domains. These effects encompass a wide range of aspects, from physical and emotional well-being [30–32] to energy poverty due to climate change [32], along with connections to homelessness and housing crises. Our research underlines the comprehensive spectrum of climate change's economic and social impacts on health. Risks and damages associated with climate-related extreme events are influenced by various factors, including exposure, vulnerability, and preparedness [22], Vulnerable populations are particularly susceptible to these socio-economic consequences due to resource and social condition disparities [31]. These findings enrich our understanding of how environmental changes affect both health and society, highlighting the necessity of holistic strategies to address these aspects of climate change for improved public health outcomes. Our study, based on the insights of medical students, has meticulously examined climate change's safety implications, including its impact on mental and social well-being, heightened psychological stress, and conflicts, which disproportionately affect marginalized populations [30]. We emphasize climate-induced housing instability and homelessness [30], alongside the emergence of environmental refugees due to migration [33]. Furthermore, our research highlights climate change's effect on urban services and resources, especially in disadvantaged communities [31], with economic disruptions impacting infrastructure and public health. Understanding these complex socio-economic dynamics is vital for comprehensive strategies to address climate change's broad impacts on health and society.

### Health effect

Climate change is recognized as the foremost 21st-century global health threat, adversely impacting essential elements including water, food, agriculture, and health through diverse mechanisms [28]. This study stands out due to its comprehensive categorization of these health consequences, reflecting the substantial focus of medical students on this subject. In contrast to a separate study by Salas, Renee N, which assessed physicians and clinical managers' awareness and opinions of climate change's health impacts [20], our study provides insights into climate change's health effects from the perspective of medical students.

Physicians, clinical managers, and leaders acknowledge climate change's impact on healthcare [20]. This study highlights medical students' concerns about climate change's effects on healthcare and health services. Rising temperatures and more frequent extreme weather events due to climate change strain health services and patient care [20]. Climate change presents challenges, including added stress on healthcare systems during hot seasons [34]. Severe heat events may lead to issues like operating room closures due to high temperatures and humidity, affecting healthcare professionals and patients and potentially disrupting healthcare services [22].

Medical students highlighted climate change's impact on healthcare professionals, revealing increased workloads, staff shortages, fatigue, psychological stress, reduced patient focus, diagnostic errors, and lower care quality. They also noted shortages of medical equipment, hospital beds, laboratory kits, rising medication demands, medication delivery disruptions, difficulties in patient transfer and assistance, limited healthcare facility access, and a heightened pandemic risk. Current concern centers on the severity and extent of future climate change impacts. Some healthcare professionals predict a growing trend in climate change's effects on healthcare services [20]. With worsening heat-related events predicted due to climate change over the next three decades, healthcare systems must prepare for increased heat-related diseases and reduce related healthcare challenges [25]. Unfortunately, health authorities have not adequately prepared for climate hazards, despite opportunities to enhance climate resilience in healthcare systems and facilities [22].

This study focuses on climate change's impact on individual health. For instance, Sarah J. Coates and colleagues have noted deteriorating health conditions and disrupted access to clean water due to climate-induced mass displacement, especially in regions like Africa [27]. Such disruptions can increase the incidence of communicable diseases [35], largely due to the vulnerability of water resources to climate stress, which makes access to clean water more challenging and costly [36]. Inadequate access to clean water, sanitation, and hygiene contributes to approximately 2.2 million annual deaths from diarrhea [24].

Medical students in this study highlight climate change's role in the emergence and spread of vector-borne diseases like malaria, dengue, and cholera due to alterations in temperature, humidity, and water availability [19, 27, 37]. Maintaining good personal hygiene to prevent water- and food-borne illnesses is also emphasized as a strategy to cope with climate change and its temperature fluctuations [19].

In this study, medical students highlighted health concerns related to climate change, including malnutrition, anemia, vitamin deficiencies, and the potential worsening of global hunger, weakened food systems, reduced nutrition, and threats to food security. Climate-related factors such as rising temperatures, erratic rainfall, and extreme weather events can impact crop yields [38]. Increased atmospheric carbon dioxide levels may also reduce the nutritional quality of certain grains and legumes, raising the risk of iron and zinc deficiencies. Climate change can also indirectly impact nutrition by influencing the spread of infectious diseases [39].

Climate change can potentially lead to malnutrition (both undernutrition and obesity) and diet-related non-communicable diseases such as diabetes and cardiovascular issues, as it adversely affects agriculture, leading to increased food and financial insecurity [39]. Furthermore, climate change significantly exacerbates the risk of famine and malnutrition by affecting agriculture, food, and water access [27]. This is especially critical in low-income countries heavily reliant on rain-fed agriculture, potentially causing significant food shortages and shifting mortality from obesity to undernutrition [39, 40]. Even high-income countries in the Western Pacific region have been affected by the repercussions of climate change on diet-related non-communicable diseases (NCDs) [40].

This study of medical students' views highlights the impact of climate change on non-communicable diseases. Higher temperatures, as noted, increase the risk of mortality from cardiovascular and heat-related diseases, exemplified by the 2003 European heatwave [28]. Additionally, climate-induced air pollution, linked to various non-communicable diseases, including respiratory ailments, lung cancer, cardiovascular events, and more, raises concerns [41]. Medical students also expressed worries about climate change potentially increasing disabilities, congenital abnormalities, and compromising the immune system [28, 41].

Climate change is linked to psychological disorders like suicide, PTSD, depression, and anxiety disorders, similar to Susan Clayton's findings [42]. Socio-economic impacts and severe weather events are the primary drivers. This study also notes a decline in hope for life and an increase in obsessive–compulsive and schizophrenic disorders as additional psychological consequences of climate change [42].

While we recognize the valuable insights gained from this study, it is essential to address the study's limitations more comprehensively. The qualitative nature of our research and the small sample size raise valid concerns about the generalizability of our findings. We acknowledge that readers should exercise caution when extrapolating the results of this qualitative study, which was conducted at a single medical school with a small number of participants. We understand the need for a more extensive discussion of these limitations to ensure that readers do not assume unwarranted certainty based on this specific study's outcomes. However, these limitations do not detract from the valuable insights gained from this specific group, confirming their strong awareness of the direct and indirect impacts of climate change on health. It underscores the need for continued education and awareness among future healthcare professionals to address the evolving challenges posed by climate change.

## Conclusions

This qualitative study based on medical students' views categorizes climate change consequences into environmental, socio-economic, and health impacts, highlighting their interconnectedness. Many of these consequences can exacerbate health outcomes. Addressing these health implications necessitates comprehensive interventions at all levels. Given the qualitative nature of this study, further research is recommended to assess the significance of newly identified health impacts related to climate change.

## Data Availability

The datasets used and/or analysed during the current study are available from the corresponding author on reasonable request.

## References

[1] Atwoli L, Baqui AH, Benfield T, Bosurgi R, Godlee F, Hancocks S (2021). Call for emergency action to limit global temperature increases, restore biodiversity, and protect health; wealthy nations must do much more, much faster. Int J Health Policy Manag.

[2] Frumkin H, Hess J, Luber G, Malilay J, McGeehin M (2008). Climate change: the public health response. Am J Public Health.

[3] Watts N, Amann M, Arnell N, Ayeb-Karlsson S, Belesova K, Boykoff M (2019). The 2019 report of the lancet countdown on health and climate change: ensuring that the health of a child born today is not defined by a changing climate. The Lancet.

[4] Hess JJ, McDowell JZ, Luber G (2012). Integrating climate change adaptation into public health practice: using adaptive management to increase adaptive capacity and build resilience. Environ Health Perspect.

[5] Maibach EW, Kreslake JM, Roser-Renouf C, Rosenthal S, Feinberg G, Leiserowitz AA (2015). Do Americans understand that global warming is harmful to human health? Evidence from a national survey. Ann Glob Health.

[6] Sarfaty M, Mitchell M, Bloodhart B, Maibach EW (2014). A survey of African American physicians on the health effects of climate change. Int J Environ Res Public Health.

[7] Xie E, de Barros EF, Abelsohn A, Stein AT, Haines A (2018). Challenges and opportunities in planetary health for primary care providers. The lancet Planetary Health.

[8] Gill M (2008). Why should doctors be interested in climate change?. BMJ.

[9] Sørensen K, Van den Broucke S, Fullam J, Doyle G, Pelikan J, Slonska Z (2012). Health literacy and public health: a systematic review and integration of definitions and models. BMC Public Health.

[10] Bugaj TJ, Heilborn M, Terhoeven V, Kaisinger S, Nagy E, Friederich H-C (2021). What do Final Year Medical Students in Germany know and think about climate change?–the climattitude study. Med Educ Online.

[11] Yang L, Liao W, Liu C, Zhang N, Zhong S, Huang C (2018). Associations between knowledge of the causes and perceived impacts of climate change: a cross-sectional survey of medical, public health and nursing students in universities in China. Int J Environ Res Public Health.

[12] Akerlof KL, Delamater PL, Boules CR, Upperman CR, Mitchell CS (2015). Vulnerable populations perceive their health as at risk from climate change. Int J Environ Res Public Health.

[13] Afkhamzadeh A, Farhadifar F, Ghotbi N, Yari A, Haydarpur M, Mohammadzadeh H (2011). Risk factors associated with borderline intelligence in schoolchildren: a case-control study. Pakistan J Med Sci.

[14] Liamputtong P, Ezzy D (2005). Qualitative research methods. Second.

[15] Elo S, Kyngäs H (2008). The qualitative content analysis process. J Adv Nurs.

[16] Graneheim UH, Lundman B (2004). Qualitative content analysis in nursing research: concepts, procedures and measures to achieve trustworthiness. Nurse Educ Today.

[17] Chowdhury P, Hashim N, Ray AK. Photocatalytic Degradation of Organic Pollutants and Airborne Pathogen in Air. Photocatalysis for Environmental Remediation and Energy Production: Recent Advances and Applications: Springer; 2023. p. 211–34.

[18] Williams JP. Long-range transport clusters, fine particulate matter (PM2. 5) and soot concentrations of air masses in Cape Town, South Africa. 2018.

[19] Chowdhury MA, Hasan MK, Islam SLU (2022). Climate change adaptation in Bangladesh: current practices, challenges and the way forward. J Climate Change Health.

[20] Salas RN (2022). The growing link between climate change and health. NEJM Catal Innov Care Deliv.

[21] Sorgho R, Jungmann M, Souares A, Danquah I, Sauerborn R (2021). Climate change, health risks, and vulnerabilities in Burkina Faso: a qualitative study on the perceptions of national policymakers. Int J Environ Res Public Health.

[22] Ebi KL, Vanos J, Baldwin JW, Bell JE, Hondula DM, Errett NA (2021). Extreme weather and climate change: population health and health system implications. Annu Rev Public Health.

[23] Rocque RJ, Beaudoin C, Ndjaboue R, Cameron L, Poirier-Bergeron L, Poulin-Rheault RA (2021). Health effects of climate change: an overview of systematic reviews. BMJ Open.

[24] Portier CJ, Tart KT, Carter SR, Dilworth CH, Grambsch AE, Gohlke J (2013). A human health perspective on climate change: a report outlining the research needs on the human health effects of climate change. J Curr Issues Global.

[25] Patel L, Conlon KC, Sorensen C, McEachin S, Nadeau K, Kakkad K (2022). Climate change and extreme heat events: how health systems should prepare. NEJM Catalyst Innov Care Deliv.

[26] Vicedo-Cabrera AM, Scovronick N, Sera F, Royé D, Schneider R, Tobias A (2021). The burden of heat-related mortality attributable to recent human-induced climate change. Nat Clim Chang.

[27] Coates SJ, Enbiale W, Davis MD, Andersen LK (2020). The effects of climate change on human health in Africa, a dermatologic perspective: a report from the International society of dermatology climate change committee. Int J Dermatol.

[28] Mousavi A, Ardalan A, Takian A, Ostadtaghizadeh A, Naddafi K, Bavani AM (2020). Climate change and health in Iran: a narrative review. J Environ Health Sci Eng.

[29] Majeed MT, Ozturk I (2020). Environmental degradation and population health outcomes: a global panel data analysis. Environ Sci Pollut Res.

[30] Bezgrebelna M, McKenzie K, Wells S, Ravindran A, Kral M, Christensen J (2021). Climate change, weather, housing precarity, and homelessness: a systematic review of reviews. Int J Environ Res Public Health.

[31] Gasper R, Blohm A, Ruth M (2011). Social and economic impacts of climate change on the urban environment. Curr Opinion Environ Sustain.

[32] Jessel S, Sawyer S, Hernández D (2019). Energy, poverty, and health in climate change: a comprehensive review of an emerging literature. Front Public Health.

[33] Shonkoff SB, Morello-Frosch R, Pastor M, Sadd J (2011). The climate gap: environmental health and equity implications of climate change and mitigation policies in California—a review of the literature. Clim Change.

[34] Rocklöv J, Forsberg B (2008). The effect of temperature on mortality in Stockholm 1998–2003: a study of lag structures and heatwave effects. Scandinavian J Pub Health.

[35] Talukder B, van Loon GW, Hipel KW, Chiotha S, Orbinski J (2021). Health impacts of climate change on smallholder farmers. One Health.

[36] Morrow G, Bowen K (2014). Accounting for health in climate change policies: a case study of Fiji. Glob Health Action.

[37] Caraka RE, Noh M, Chen R-C, Lee Y, Gio PU, Pardamean B (2021). Connecting climate and communicable disease to penta helix using hierarchical likelihood structural equation modelling. Symmetry.

[38] Romanello M, McGushin A, Di Napoli C, Drummond P, Hughes N, Jamart L (2021). The 2021 report of the lancet countdown on health and climate change: code red for a healthy future. The Lancet.

[39] Fanzo JC, Downs SM (2021). Climate change and nutrition-associated diseases. Nat Rev Dis Primers.

[40] Springmann M, Mason-D'Croz D, Robinson S, Garnett T, Godfray HCJ, Gollin D (2016). Global and regional health effects of future food production under climate change: a modelling study. The Lancet.

[41] Manisalidis I, Stavropoulou E, Stavropoulos A, Bezirtzoglou E (2020). Environmental and health impacts of air pollution: a review. Front Pub Health.

[42] Clayton S (2021). Climate change and mental health. Curr Environ Health Rep.

